# Synthesis of Samarium‐incorporated X‐ray‐sensitive nanoparticles and hydrogels: Diverse applications in radiation monitoring and dynamic information display

**DOI:** 10.1002/smo2.70048

**Published:** 2026-04-27

**Authors:** Zetong Zhang, Yujie Chen, Yuanyao Chen, Leipeng Li, Yonggang Wu, Yanmin Yang, Hailei Zhang

**Affiliations:** ^1^ College of Chemistry & Materials Science Hebei University Baoding China; ^2^ College of Physics Science and Technology Hebei University Baoding China

**Keywords:** afterglow, halloysite, hydrogel, luminescence, nanomaterial, X‐ray

## Abstract

With the advancement of X‐ray‐activated persistent luminescent phosphors, the exploration of smart materials with tunable luminescence and afterglow properties is a promising topic. In this study, the red‐emission X‐ray scintillators, Samarium(Sm^3+^)‐doped NaLuF_4_, with regular micromorphology and well‐controlled afterglow property were synthesized under mild conditions. Both of Sm^3+^‐doped *α*‐NaLuF_4_ and *β*‐NaLuF_4_, abbreviated as *α*‐NaLuF_4_:*x*Sm^3+^ and *β*‐NaLuF_4_:*y*Sm^3+^ (*x*, *y* = 1, 3%, 5%, 10%, and 15%), respectively, emit the maximum emission peak at 598 nm (4G_5/2_ → 6H_7/2_) under X‐rays, while only Sm^3+^‐doped *β*‐NaLuF_4_ exhibits remarkable afterglow ability. The optimal luminescent property can be obtained when the doping rate is fixed at 3%. Then *β*‐NaLuF_4_:3%Sm^3+^ was surface modified and incorporated into the chemically crosslinked hydrogels to endow X‐ray‐activated luminescence or afterglow abilities in the obtained hybrid hydrogel. With the self‐healing ability, the *β*‐NaLuF_4_:3%Sm^3+^‐incorporated hydrogel can be further programmed with other types of hydrogels to enable tunable emission behaviors and time‐dependent color changes. By taking advantage of these features, the hydrogels were programmed as a display panel that can be used for extra safety information encryption.

## Graphical abstract

Sm^3+^‐doped *α*‐NaLuF_4_ and *β*‐NaLuF_4_ were synthesized. The X‐ray‐activated persistent luminescence abilities and afterglow properties can be well controlled. Sm^3+^‐doped *β*‐NaLuF_4_ exhibits remarkable afterglow ability. The hexagonal *β*‐NaLuF_4_:3%Sm^3+^‐incorporated hydrogel can be further programmed with other types of hydrogels to enable tunable emission behaviors, time‐dependent color changes, and extra safety information encryption.

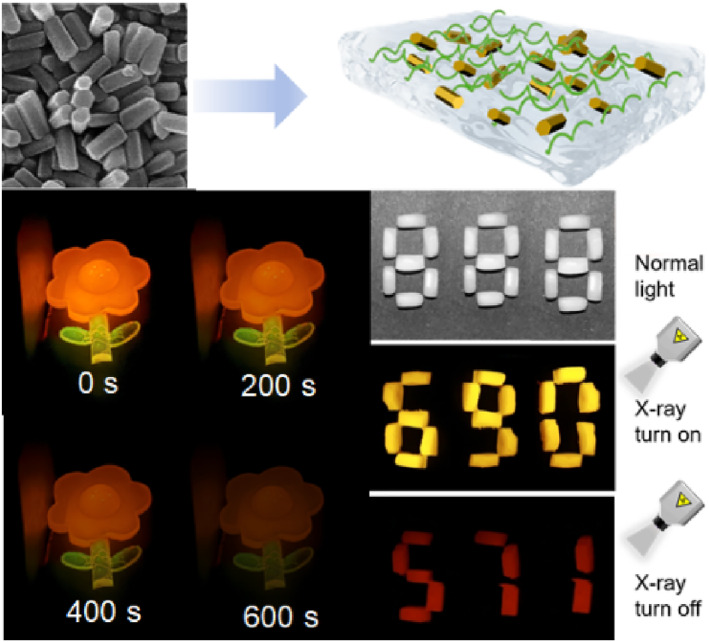

## 1 INTRODUCTION

Persistent luminescent phosphors continuously stimulate the extensive research interest in the fields including optics, physics, chemistry, industry, crystallography, and even biomedicine, which can store energy under irradiation and then continuously deliver luminescence for several minutes, hours, or even for days after turning off the irradiation.[^1^, ^2^] The applications of persistent luminescent phosphors have ranged from civil uses, that is, illumination,^[3]^ decoration,^[4]^ displays,^[5]^ etc., to broad scientific fields, including medical image,^[6]^ photodynamic therapy,^[7]^ information encryption,^[8]^ energy,^[9]^ probe,^[10]^ and sensors.^[11]^ Besides traditionally‐used irradiations, including ultraviolet,^[12]^ visible light,^[13]^ and near‐infrared light,^[14]^ X‐rays can serve as a high‐energy excitation for developing persistent luminescent phosphors with more mysterious optical phenomena.[^15^, ^16^] As a result, X‐ray‐activated persistent luminescent phosphors have raised a lot of attention in the past decade.^[17]^ Nevertheless, the current generation of X‐ray‐activated persistent luminescent phosphor materials are mainly large crystals, grown under harsh conditions and prepared by mechanical grinding, which lack microstructure modulation, tunable afterglow properties, and dispersibility in solution for thin‐film processing.[^18^, ^19^]

Many attempts have been made to develop X‐ray‐activated persistent luminescent phosphors with well‐designed microstructures and tunable afterglow properties. Pioneering works have been reported by several groups. Liu's group demonstrated ultralong‐lived X‐ray trapping by synthesizing terbium (Tb^3+^)‐doped core–shell nanoscintillators, which can release >30 days of persistent luminescence.^[20]^ Xia's group developed X‐ray‐activated persistent luminescent phosphors with tunable photophysical properties by controlling Mn^2+^ site occupation manipulation and heterovalent substitution.^[21]^ Our group reported a series of X‐ray‐activated, lanthanide‐doped nanoparticles with diverse emission abilities ranging from ultraviolet and visible to near‐infrared window.[^22^, ^23^] Up to now, though several underlying mechanisms have been launched to explain the X‐ray‐induced persistent luminescence, such as the hole trapping‐detrapping model, the electron trapping‐detrapping model, and the quantum tunneling model,[^24^, ^25^, ^26^] some key points are still in doubt. To our best knowledge, persistent luminescence is governed by the following aspects: whether the excitation can effectively store energy, whether the heat can effectively release charge carriers, and whether the luminescent center can effectively bind charge carriers and produce emissions.[^27^, ^28^] We proposed a mechanism for the persistent luminescence behaviors of trivalent lanthanides in a previous study.^[29]^ As isoelectronic traps, the trivalent lanthanides are expected to bind excitons. The binding ability is not only related to the inherent arrangement of the electrons but also to the extrinsic anion coordination and cation substitution in the host lattices. Hexagonal‐phased lattices are suitable for achieving energy transfer and energy migration, while a cubic lattice with eight coordinated holes easily contributes to nonradiative quenching through reduced migration of the exciton energy.[^30^, ^31^] We hypothesize that the controllable construction of cubic and hexagonal lattices is the key to achieving tunable afterglow properties of X‐ray‐activated persistent luminescent phosphors.

Herein, we synthesized a series of Samarium(Sm^3+^)‐doped NaLuF_4_ scintillators under mild conditions. The lattice type can be well‐controlled by facilely regulating the pH values, to afford *α*‐NaLuF_4_:*x*Sm^3+^ and *β*‐NaLuF_4_:*y*Sm^3+^ with cubic and hexagonal lattices, respectively, where *x* and *y* represent the doping rate of Sm^3+^. We investigated the influence of structural parameters and the doping rate of Sm^3+^‐doped NaLuF_4_ scintillators on their X‐ray‐excited luminescence intensity and afterglow properties. The obtained phosphors were surface modified to endow them with desirable water‐dispersibilities and then incorporated into the chemically crosslinked hydrogels with dynamic covalent bonds. The mechanical properties, as well as self‐healing abilities, were carefully investigated. Information encryption can be facilely achieved by programming the above‐mentioned hydrogel pieces in a panel via self‐healing properties.

## 2 RESULTS AND DISCUSSIONS

### 2.1 Synthesis and structural characterization

**FIGURE 1:**
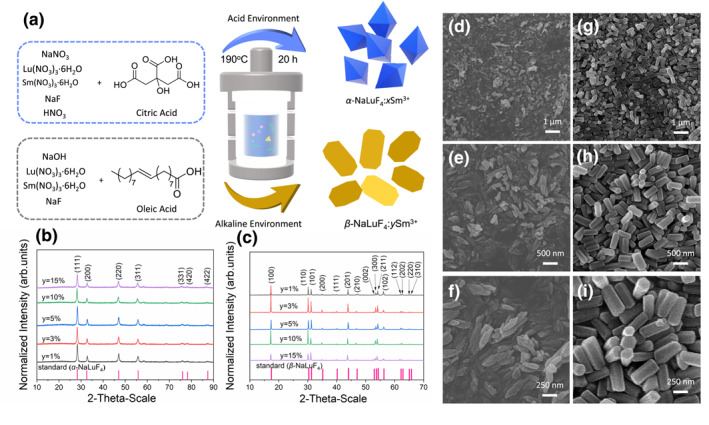
Synthesis and structural characterization of *α*‐NaLuF_4_:*x*Sm^3+^ and *β*‐NaLuF_4_:*y*Sm^3+^. (a) Synthesis route to solvothermally prepare *α*‐NaLuF_4_:*x*Sm^3+^ and *β*‐NaLuF_4_:*y*Sm^3+^. (b) XRD patterns of the obtained *α*‐NaLuF_4_:*x*Sm^3+^ (*x* = 1, 3%, 5%, 10%, and 15%) and standard (JCPDS no. 27–0725). (c) XRD patterns of the obtained *β*‐NaLuF_4_:*y*Sm^3+^ (*y* = 1, 3%, 5%, 10%, and 15%) and standard (JCPDS no. 27–0726). (d–f) SEM images of *α*‐NaLuF_4_:3%Sm^3+^. (g–i) SEM images of *β*‐NaLuF_4_:*y*Sm^3+^.

A unit cell diagram of cubic *α*‐NaLuF_4_ was drawn referring to the crystal structure database and depicted in Supporting Information S1: Figure S1, accompanied by the crystal systems and space‐groups. For the cubic phase of *α*‐NaLuF_4_, the cubic unit cell parameter is approximately 5.471 Å. The hexagonal *β*‐NaLuF_4_ with constants of a = 5.901 Å and c = 3.453 Å is based on JCPDS 27–0726. Here, the ionic radii of cations are Sm^3+^: ∼0.958 Å and Lu^3+^: ∼0.848 Å, respectively, which enables the opportunity of replacing Lu^3+^ by Sm^3+^ ion in cubic *α*‐NaLuF_4_ and hexagonal *β*‐NaLuF_4_ lattices on account of their same valence states and similar ionic radii. The Samarium‐incorporated phosphors, *α*‐NaLuF_4_:*x*Sm^3+^ and *β*‐NaLuF_4_:*y*Sm^3+^, were synthesized via a hydrothermal reactor under different pH values (Figure 1a). A weak acid environment was beneficial to afford the cubic phase product, whereas the hexagonal *β*‐NaLuF_4_:*y*Sm^3+^ was synthesized under an alkaline environment. The yielded products were carefully characterized by XRD, XPS, and SEM.

Figure 1b shows the XRD patterns of *α*‐NaLuF_4_:*x*Sm^3+^. The sample exhibited pure *α*‐NaLuF_4_ (JCPDS 27–0725) when the Sm^3+^ content (*x*) ranged from 1%, 3%, 5%, 10%, to 15%. As for *β*‐NaLuF_4_:*y*Sm^3+^ (*y* = 1, 3%, 5%, 10%, and 15%) shown in Figure 1c, strong diffraction peaks appeared at 17.3°, 30.1°, 31.2°, 44.0°, and 52.9° are assigned to the (100), (110), (101), (201), and (002) planes of *β*‐NaLuF_4_, respectively. Weaker diffraction peaks between 70 and 80° are attributed to the (311), (212), and (302) planes. The diffraction peaks of *β*‐NaLuF_4_:*y*Sm^3+^ are in accordance with the JCPDS no. 27–0726. No obvious shift or change is observed in the XRD patterns after doping Lu^3+^ by Sm^3+^ in the NaLuF_4_ lattice. This can be mainly attributed to the very close ionic radii of Sm^3+^ and Lu^3+^, both belonging to the trivalent lanthanide metal ions. Thus, the replacement of Lu^3+^ by Sm^3+^ did not significantly alter the crystal structure or interplanar spacing, also evidenced by the Rietveld refinement results (Supporting Information S1: Figure S2). Therefore, the positions and shapes of the diffraction peaks were almost unchanged, confirming the successful doping of Sm^3+^ into the NaLuF_4_ host lattice without the formation of impurity phases.

The SEM images of *α*‐NaLuF_4_:3%Sm^3+^ displayed irregular particles in Figure 1d–f. The high surface energy of cubic nanocrystals usually gives rise to unstable energy during the synthesis process and makes them prone to agglomeration or morphological transformation.[^32^, ^33^] The SEM images depicted in Figure 1g–i showed that *β*‐NaLuF_4_:%Sm^3+^ were found to be uniform hexagonal prisms with a diameter of <1 μm, which matches the findings of XRD patterns. The uniform distribution and micromorphological structures inspired us to further explore their X‐ray‐excited luminescence properties.

**FIGURE 2:**
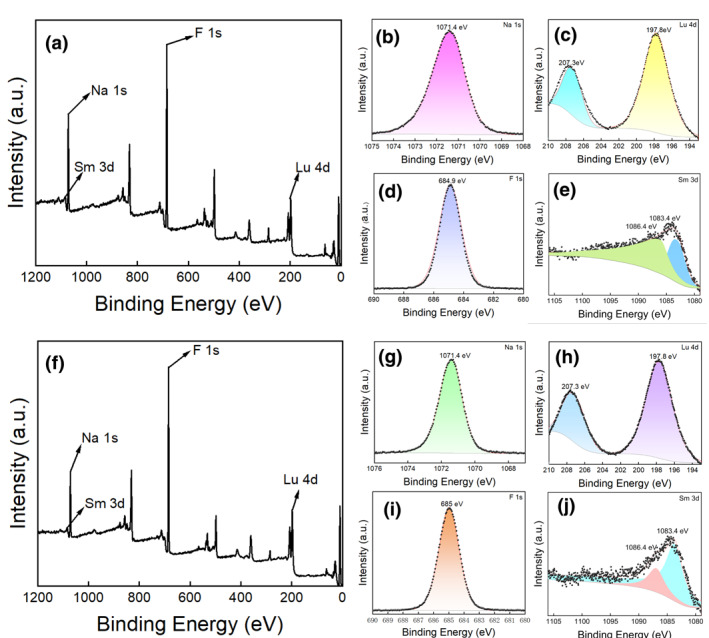
XPS patterns. (a) XPS pattern of *α*‐NaLuF_4_:3%Sm^3+^. (b) Na 1s region. (c) Lu 4d region. (d) F 1s region. (e) Sm 3d region. (f) XPS pattern of *β*‐NaLuF_4_:*y*Sm^3+^. (g) Na 1s region. (h) Lu 4d region. (i) F 1s region. (j) Sm 3d region.

XPS was used to reveal the chemical compositions of *α*‐NaLuF_4_:3%Sm^3+^ (Figure 2a) and *β*‐NaLuF_4_:3%Sm^3+^ (Figure 2f). The detailed results are shown in Figure 2. The presence of sodium was confirmed by the Na 1s peaks at 1071.4 eV in both the XPS spectra of *α*‐NaLuF_4_:3%Sm^3+^ (Figure 2b) and *β*‐NaLuF_4_:3%Sm^3+^ (Figure 2g). The peaks at 207.3 (Figure 2c) and 197.8 eV (Figure 2h) were attributed to the Lu 4d peaks, demonstrating the presence of lutetium in *α*‐NaLuF_4_:3%Sm^3+^ and *β*‐NaLuF_4_:3%Sm^3+^. The F 1s peaks at 684.9 eV can be clearly observed at 684.9 (Figure 2d) and 685.0 eV (Figure 2i), indicating the presence of fluorine. Weaker peaks at 1064.4 and 1083.4 eV (Figure 2e,g) were attributed to the Sm 3d signals. The chemical compositions were in accordance with the expected products.

### 2.2 Spectral properties

**FIGURE 3:**
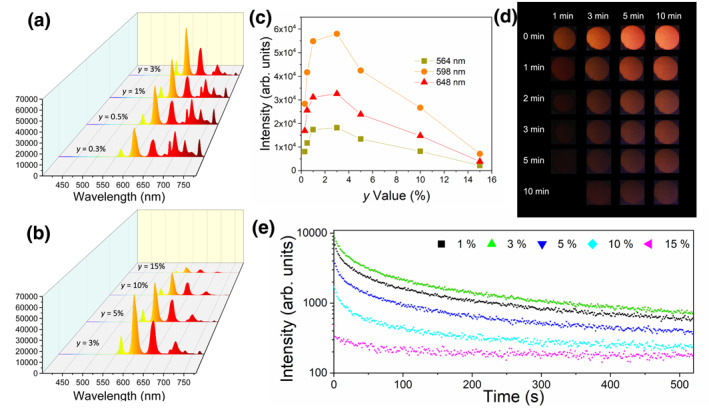
Optical properties. (a) X‐ray‐excited luminescence emission spectra of *β*‐NaLuF_4_:*y*Sm^3+^ (*y* = 0.3, 0.5%, 1%, and 3%). (b) X‐ray‐excited luminescence emission spectra of *β*‐NaLuF_4_:*y*Sm^3+^ (*y* = 3, 5%, 10%, and 15%). (c) Plots of X‐ray‐excited luminescence intensity at 564, 598, and 648 nm *vers* different *y* values. (d) Photographs of four sample (NaLuF_4_:3%Sm^3+^) discs taken at different afterglow times (1–10 min) after irradiation with X‐rays for 1–10 min. The discs were placed on a black plate surface for imaging in a dark room. (e) Afterglow intensity from *β*‐NaLuF_4_:3%Sm^3+^ monitored at 598 nm as a function of time.

X‐ray‐excited luminescence emission spectra of the Sm^3+^‐doped samples are summarized in Figure 3a,b. The spectra exhibited three main peaks at 564, 598, and 648 nm, originating from the characteristic transitions of 4G_5/2_ → 6H_5/2_, 4G_5/2_ → 6H_7/2_ and 4G_5/2_ → 6H_9/2_, respectively.^[34]^ The intensity of the maximum emission peak, located at 598 nm, increased as the doping ratio increased from 0.1% to 3% (Figure 3c). The result indicated that the substitution of Lu^3+^ with Sm^3+^ in the lattice was beneficial to enhance the radioluminescence. Further increase of Sm^3+^ concentration contributed to a decreasing trend in the radioluminescence intensity owing to the concentration quenching effect, which is a commonly believed phenomenon for lanthanide‐based phosphor.[^35^, ^36^] Moreover, the tendencies of the emission peak intensity at 564 and 648 nm, assigned to 4G_5/2_ → 6H_5/2_ and 4G_5/2_ → 6H_9/2_ transitions, are the same as that of the maximum emission peak. In addition to the above‐discussed main emission peaks by Sm^3+^, some additional peaks ranging from 700 to 820 nm were presented. Different from the above‐discussed peaks, the intensities of the peaks ranging from 700 to 820 nm decrease as the Sm^3+^ concentration increases from 0.3% to 15%. Such emissions were rarely reported in Sm^3+^‐doped phosphors, but in accordance with the emission peaks from Sm^2+^.^[37]^ Among the rare earth ions, the Sm^3+^ to Sm^2+^ conversion is of particular interest.^[38]^ The conversion is very easy to take place with the exposure to X‐ray irradiation, especially in low doping concentrations,^[39]^ owing to the inadequate binding from the host lattices. As a result, the peaks ranging from 700 to 820 nm observed in the X‐ray‐excited luminescence emission spectrum should be ascribed to ^5^D_0_→^7^F_J_ transitions of Sm^2+^.

Interestingly, *β*‐NaLuF_4_:*y*Sm^3+^ samples exhibited typical X‐ray‐activated persistent luminescence behaviors. Take *β*‐NaLuF_4_:3%Sm^3+^ as an example, the emitted red luminescence was discernible to the naked eyes for more than 10 min after X‐ray irradiation for >3 min (Figure 3d). The persistent luminescence decay curves collected at 598 nm after X‐ray irradiation were recorded, as shown in Figure 3e. The afterglow intensity decreased rapidly in the first 100 s and then decayed tardily. Clearly, *β*‐NaLuF_4_:3%Sm^3+^ with a uniform hexagonal morphology exhibited better afterglow properties than the other materials. The thermoluminescence (TL) spectra were recorded at 598 nm to investigate the trap distribution of *β*‐NaLuF_4_:3%Sm^3+^ (Supporting Information S1: Figure S3). After excitation by X‐ray for 5 min, the TL curve of *β*‐NaLuF_4_:3%Sm^3+^ was monitored at a heating rate of 5°C/min. There are two broad peaks within the 273–700K temperature range. Based on this result, the trap‐depth, *E*, can be calculated by using the following empirical Urbach's formula:

(1) $$E=\frac{T_{m}}{500}$$

where *T*_*m*_ is the temperature corresponding to the peak value of the TL spectrum. Based on Equation (1), *β*‐NaLuF_4_: 3%Sm^3+^ possesses two trap depths: the shallow trap depth *E*_1_ at low temperature is approximately 0.58 eV, and the deep trap depth *E*_2_ at high temperature is about 0.90 eV.

**FIGURE 4:**
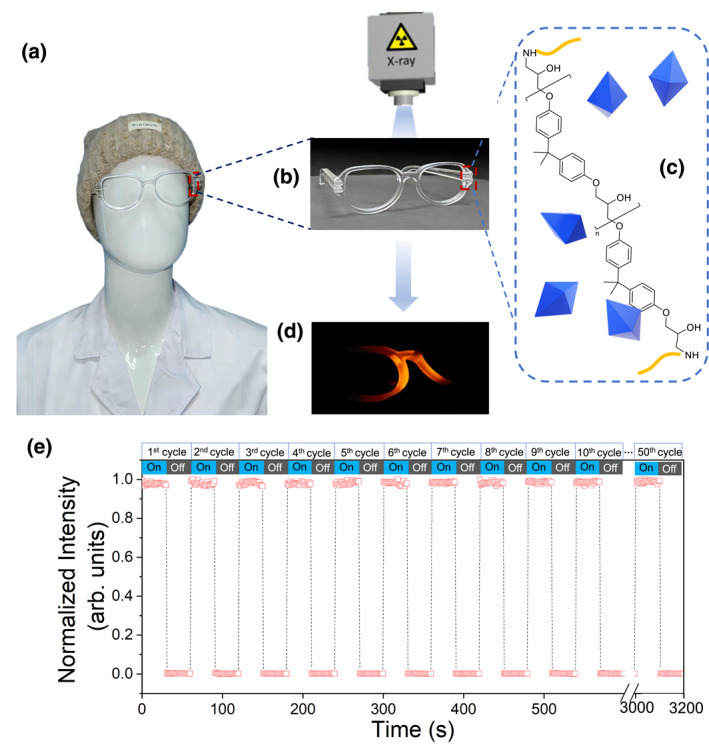
Fabricating X‐ray‐sensitive composite and its application in radiation monitoring. (a) Photograph of the mannequin wearing a self‐made eyeglass frame made by *α*‐NaLuF_4_:3%Sm^3+^‐incorporated epoxy resin composite. (b) Photograph of the self‐made eyeglass frame under day light. (c) Chemical composition of the eyeglass frame. (d) Photograph of the self‐made eyeglass frame under X‐rays. (e) In situ measurement of the luminescence intensity of *α*‐NaLuF_4_:3%Sm^3+^‐incorporated epoxy resin composite under X‐ray with on‐off cycles.

Like *β*‐NaLuF_4_:3%Sm^3+^, *α*‐NaLuF_4_:3%Sm^3+^ also displayed the optimal X‐ray‐activated emission ability among all *α*‐NaLuF_4_:*x*Sm^3+^ (*x* = 1, 3%, 5%, 10%, and 15%) samples. Three main peaks at 564, 598, and 648 nm can also be detected, but in the absence of persistent luminescence. The cubic lattice was found to easily contribute to nonradiative quenching through reduced migration of the exciton energy. Then, we incorporated the obtained *α*‐NaLuF_4_:3%Sm^3+^ into an epoxy resin and fabricated it into an eyeglass frame with good transparency (Figure 4a–c). It can emit visible red light under low‐energy X‐ray (30 kV), which is much lower than that used in brachytherapy. Moreover, the sensitivity of *α*‐NaLuF_4_:3%Sm^3+^‐incorporated composite towards X‐ray was investigated based on on‐off cycles (Figure 4e). It exhibited a synchronous emission behavior following the on‐off switching of X‐ray irradiation. The intensity and sensitivity can be maintained for more than 50 cycles. This developed eyeglass frame provided opportunities for in situ radiation exposure monitoring. Though up to now, diverse wearable tools have been made to monitor the radiation exposure, for example, wristband,^[40]^ clothing,^[41]^ electronics,^[42]^ etc., the red luminescence emitted from the eyeglass frame can be the most observable way to people.

### 2.3 Fabricating hydrogels

**FIGURE 5:**
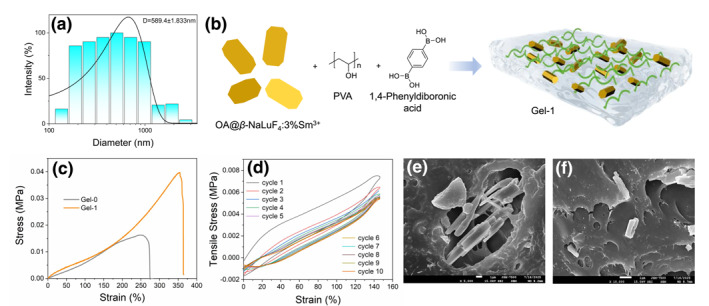
Synthesis, characterization, and mechanical properties of OA@*β*‐NaLuF_4_:3%Sm^3+^‐incorporated hydrogel (Gel‐1). (a) Size distribution of OA@*β*‐NaLuF_4_:3%Sm^3+^ in the aqueous phase. (b) Synthetic route to Gel‐1. (c) Stress‐strain curves of tensile testing. (d) Fatigue resistance of Gel‐1 investigated at 150% strain for 10 cycles. (e, f) SEM image of Gel‐1.

The obtained *β*‐NaLuF_4_:3%Sm^3+^ was surface modified with oleic acid (OA) by referring to our previous study.^[43]^ The water dispersibility of the yielded product, OA@*β*‐NaLuF_4_:3%Sm^3+^, was comprehensively improved, as evidenced by a dynamic light scattering (DLS) spectrometer from Brookhaven BI‐200SM Goniometer equipped with a 633 nm laser. The effective diameter of the obtained OA@*β*‐NaLuF_4_:3%Sm^3+^ is determined as 589.4 nm with a polydispersity index (PDI) of 0.278 in water (Figure 5a) by using a Laplace inversion program. Inspired by the desirable water‐dispersibility, the obtained OA@*β*‐NaLuF_4_:3%Sm^3+^ was incorporated into a chemically crosslinked hydrogel made of PVA and 1,4‐phenyldiboronic acid (Figure 5b).

Figure 5c shows the stress‐strain tensile curves of the hydrogel samples. Gel‐0 and Gel‐1 represent the 1,4‐phenyldiboronic acid‐crosslinked PVA hydrogels with the absence and presence of OA@*β*‐NaLuF_4_:3%Sm^3+^, respectively. The incorporation of OA@*β*‐NaLuF_4_:3%Sm^3+^ in the hydrogel was beneficial for increasing the elongation at break compared to the pristine hydrogel. Gel‐0 exhibited an elongation at break of ca. 250% with a fracture strength of 16.3 KPa, whereas the elongation at break of Gel‐1 increased to ca. 354%, together with an increased fracture strength of 39.7 KPa. The nanohexagonal OA@*β*‐NaLuF_4_:3%Sm^3+^ with a large length‐diameter ratio contributed to improved tensile property.^[44]^ The compression performance test was also conducted to afford stress‐strain curves of Gel‐0 and Gel‐1 shown in Supporting Information S1: Figures S4 and S5. As for Gel‐1, the modulus of compression was calculated as 0.17 kPa, which was obviously higher than Gel‐0 (0.11 kPa). Fatigue resistance of Gel‐1 was investigated at 150% strain for 10 cycles (Figure 5d). No visible fracture was found on the hydrogel piece after the completion of 10 fatigue load cycles. As the cycle number increases from 1 to 10, the Young's modulus decreases and remains around ca. 60% as compared to pristine Gel‐1 until the end of the test.

The SEM images shown in Figure 5e,f revealed the micromorphology of Gel‐1, which displayed porous structures in accordance with the well‐reported hydrogel samples.[^45^, ^46^] The hexagonal nanoparticles were clearly visible in the image with high magnification, demonstrating the successful incorporation of the OA@*β*‐NaLuF_4_:3%Sm^3+^ in the hydrogel matrix.

**FIGURE 6:**
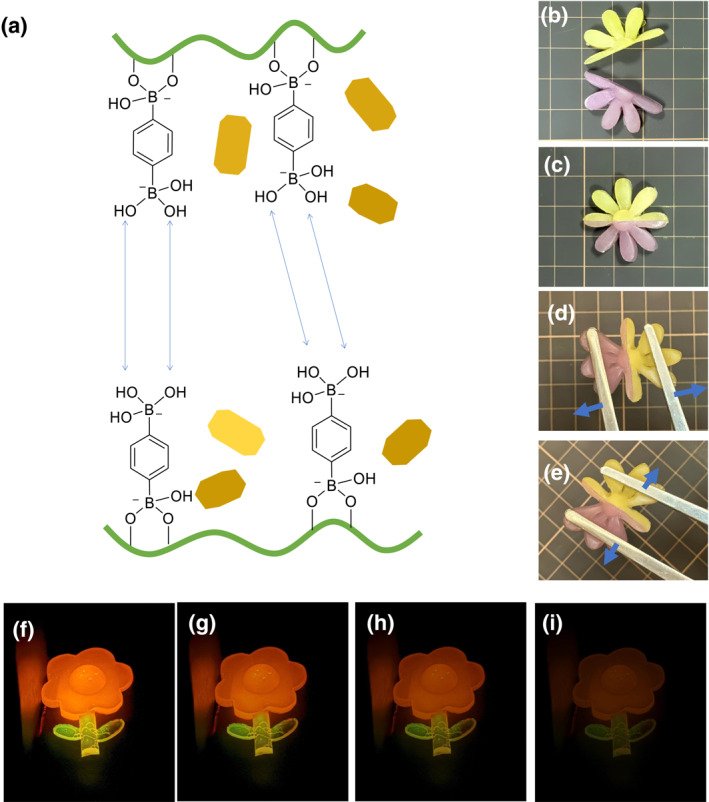
Self‐healing properties and programmed display panel. (a) Photo of the as‐prepared flower‐like hydrogel sample. (b) Separated hydrogel pieces. (c) Re‐jointed hydrogel pieces. (d) Self‐healed hydrogel. (e) Self‐healing mechanism. (f) Programmed plant‐like hydrogel with exposure to X‐ray. The top part comes from OA@*β*‐NaLuF_4_:3%Sm^3+^‐incorporated hydrogel (Gel‐1) and the bottom part comes from HNTs@YF_3_:Tb^3+^‐incorporated hydrogel (Gel‐2). (g) Programmed plant‐like hydrogel after ceasing X‐ray for 200 s. (h) Programmed plant‐like hydrogel after ceasing X‐ray for 400 s. (i) Programmed plant‐like hydrogel after ceasing X‐ray for 600 s.

The arylboronic acid moieties in the polymer matrix are capable of reacting with other diol groups on PVA chains (Figure 6a),^[47]^ which endows the as‐prepared hydrogels with dynamic self‐healing property. The OA@*β*‐NaLuF_4_:3%Sm^3+^‐containing hydrogel (Gel‐1) can afford a rough micromorphology of the cracked surface, which is beneficial for achieving a desired self‐healing property.

To test this point, stained hydrogel samples were prepared. Two Gel‐1 pieces were stained with 0.01 wt% yellow or purple liquid pigment, respectively. Each of the stained hydrogel samples was cut into two equant pieces (Figure 6b). The half‐hydrogel pieces from the yellow and purple samples were joint together for 5 min without any external forces at room temperature (Figure 6c). The healed hydrogel sample can be stretched without obvious breakage or fracture. The desired self‐healing property enables further programming of multiple‐color display panels. Halloysite nanotube‐based X‐ray‐activated persistent luminescent phosphor, HNTs@YF_3_:Tb^3+^, was synthesized based on our previous study^[23]^ and then incorporated in the chemically crosslinked hydrogel. The yielded Gel‐2 can emit green luminescence and afterglow after exposure to X‐rays. Gel‐2 was further fabricated as a branched shape and then attached to the flower‐like Gel‐1 via the self‐healing behaviors. When exposed to X‐rays, the programmed composite displayed multi‐color emission behaviors. The top part comes from Gel‐1 emitting red luminescence, while the bottom part comes from Gel‐2 emitting green light (Figure 6f). The composite presented a plant‐like version under X‐ray. After ceasing the X‐rays, the afterglow can also be observed by naked eyes for 600 s (Figure 6g–i).

**FIGURE 7:**
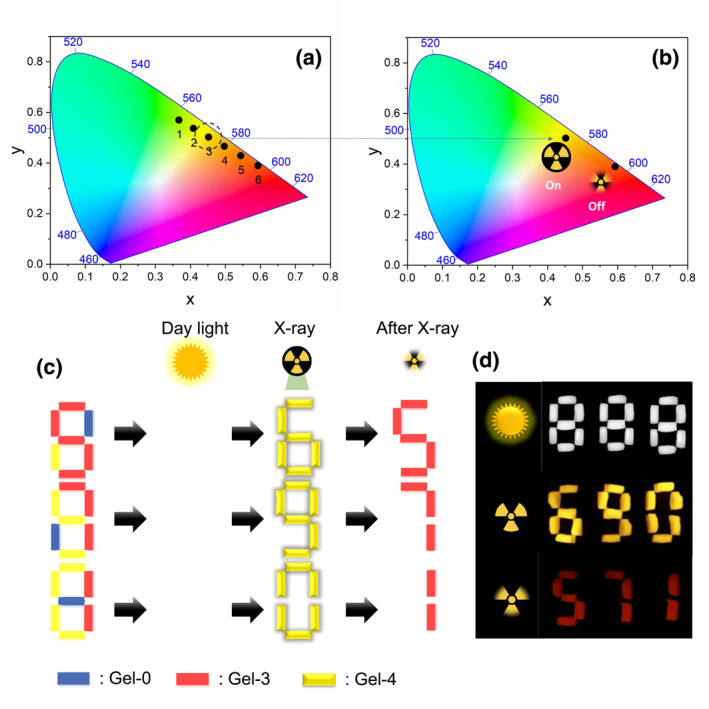
Fabricate the emission‐tunable scintillating hydrogel with time‐dependent color change and information encryption studies. (a) CIE 1931 chromaticity diagram of the emission‐tunable scintillating hybrid hydrogel doped with different feed ratio between HNTs@*α*‐NaLuF_4_:Tb^3+^ and OA@*β*‐NaLuF_4_:3%Sm^3+^ (1:0, 1:4, 2:3, 3:2, 4:1, and 0:1 for case 1, 2, 3, 4, 5, and 6, respectively). (b) CIE 1931 chromaticity diagram of Gel‐3 with the exposure to X‐rays and after ceasing the X‐rays for 200 s (c) Scheme of programming encrypted and faked information based on assembling the hydrogel pieces. (d) Photograph of the different types of information of the programmed hydrogels under daylight, X‐ray, and the cessation of X‐ray.

To fabricate the emission‐tunable scintillating hydrogel with time‐dependent color change, an HNTs‐based X‐ray‐activated luminescent nanocomposite (HNTs@*α*‐NaLuF_4_:Tb^3+^) in the absence of afterglow property was synthesized according to our previous study. Figure 7a showcases the emission properties of emission‐tunable scintillating hybrid hydrogel doped with different feed ratios between HNTs@*α*‐NaLuF_4_:Tb^3+^ and OA@*β*‐NaLuF_4_:3%Sm^3+^ (1:0, 1:4, 2:3, 3:2, 4:1, and 0:1 for cases 1, 2, 3, 4, 5, and 6, respectively). The peak intensity of 544 nm (^5^D_4_‐^7^F_5_) associated with Tb^3+^ decreased as the content of OA@*β*‐NaLuF_4_:3%Sm^3+^ increased, whereas the peak intensities at 564 (4G_5/2_ → 6H_5/2_), 598 (4G_5/2_ → 6H_7/2_), and 648 nm (4G_5/2_ → 6H_9/2_) enhanced. As a result, the emission‐tunable behavior (green → yellow → red) can be visually observed in the hybrid hydrogels doped with different feed ratios between HNTs@*α*‐NaLuF_4_:Tb^3+^ and OA@*β*‐NaLuF_4_:3%Sm^3+^. Benefiting from the difference in afterglow abilities, time‐dependent color changes can also be programmed. Taking the hybrid hydrogel with a feed ratio between HNTs@*α*‐NaLuF_4_:Tb^3+^ and OA@*β*‐NaLuF_4_:3%Sm^3+^ as 2:3 (Gel‐3) as an example, it can emit yellow luminescence upon exposure to X‐rays. After ceasing the X‐rays, the red emission can continue for seconds owing to the afterglow generated from OA@*β*‐NaLuF_4_:3%Sm^3+^, whereas the green emission rapidly decayed within milliseconds because of the absence of the afterglow ability of HNTs@*α*‐NaLuF_4_:Tb^3+^. Thus, a color change from yellow to red can be achieved accompanying switching off the X‐rays. Following this method, the color change‐based multiple‐level information encryption can be facilely programmed.

Figure 7c showcases the programming of different types of hydrogel samples, including Gel‐0, Gel‐3, and Gel‐4. Gel‐4 was prepared by incorporating OA@*α*‐NaLuF_4_:3%Sm^3+^ (Supporting Information S1: Figure S6) and HNTs@*α*‐NaLuF_4_:Tb^3+^ in the chemically‐crosslinked hydrogel, which displayed no afterglow ability after ceasing X‐rays. Gel‐0, Gel‐3, and Gel‐4 were fabricated into “8”‐like shapes. Each hydrogel piece exhibited no difference from the others and the assembled panel displayed the information of “888” under daylight (Figure 7d). When exposed to X‐rays, the information of “690” emitting yellow luminescence can be nakedly observed. Though Gel‐3 and Gel‐4 contain different kinds of phosphors, the hydrogel bars from Gel‐3 and Gel‐4 displayed the same luminescent behavior with exposure to X‐ray. After the cessation of X‐ray, the color changed from yellow to red. Simultaneously, the encrypted information, “571”, appeared and can be visibly observed. Such an interesting and color‐adjustable information encryption programming may pave a novel path for designing advanced anticounterfeiting flexible materials with broader applications.

In contrast to most scintillator materials reported in the literature,[^48^, ^49^, ^50^, ^51^] which usually require ultrahigh‐temperature preparation conditions (>1000°C), Sm^3+^‐doped NaLuF_4_ scintillators in this work were synthesized via a mild and low‐energy‐consumption method in the aqueous phase. Moreover, the crystal phase of the product can be effectively tuned by simply adjusting the pH value during synthesis, which further enables the facile control on the afterglow properties. In addition, the as‐prepared Sm^3+^‐doped NaLuF_4_ scintillator exhibits red emission, which is quite suitable for practical applications including radiation leakage monitoring and information anti‐counterfeiting. These advantages in synthesis conditions, phase controllability, afterglow modulation, and optical performance make it a very competitive candidate compared with conventional scintillator materials.

## 3 CONCLUSION

In summary, Sm^3+^‐doped NaLuF_4_ scintillators were successfully synthesized. Both of *α*‐NaLuF_4_:*x*Sm^3+^ and *β*‐NaLuF_4_:*y*Sm^3+^ can emit orange‐red light under excitation by X‐ray with the maximum emission peak at 598 nm (4G_5/2_ → 6H_7/2_), while only Sm^3+^‐doped *β*‐NaLuF_4_ exhibits remarkable afterglow ability. The optimal luminescent property can be obtained when the doping rate is fixed at 3%. The obtained *α*‐NaLuF_4_:3%Sm^3+^ and *β*‐NaLuF_4_:3%Sm^3+^ were surface modified with OA to afford the products with good dispersibility in water and polymeric matrix. The effective diameters of the obtained OA@*α*‐NaLuF_4_:3%Sm^3+^ and OA@*β*‐NaLuF_4_:3%Sm^3+^ were determined as 589.4 and 753.8 nm, with PDI values of 0.278 and 0.311, respectively. OA@*α*‐NaLuF_4_:3%Sm^3+^ was incorporated into the epoxy resin to fabricate an eyeglass frame with X‐ray‐sensitive luminescence for in situ radiation exposure monitoring. OA@*β*‐NaLuF_4_:3%Sm^3+^ was incorporated into the 1,4‐phenyldiboronic‐crosslinked hydrogels with desirable self‐healing property and fatigue resistance ability. The hydrogels were further programmed as diverse panels for dynamic display and information encryption. We foresee that such systems will inspire the development of smart materials for exposure monitoring, multiple anticounterfeiting, and multi‐dimensional programming.

## CONFLICT OF INTEREST STATEMENT

The authors declare no conflicts of interest.

## ETHICS STATEMENT

This study did not involve human participants or animal experiments.

## Supporting information

Supporting Information S1

## Data Availability

The data that supports the findings of this study are available in the Supporting Information of this article.
