# Supplementary material for: Synthesis of Samarium‐incorporated X‐ray‐sensitive nanoparticles and hydrogels: Diverse applications in radiation monitoring and dynamic information display
Source: Smart Mol. 2026 Apr 27:e70048. Online ahead of print. doi: 10.1002/smo2.70048 (PMC13399032; doi:10.1002/smo2.70048)
Supplement: Supplementary file 1 — Supporting Information S1 [file SMO2-9999-0-s001.doc]

Supporting Information

Synthesis of Samarium-Incorporated X‑Ray-Sensitive Nanoparticles and Hydrogels: Diverse Applications in Radiation Monitoring and Dynamic Information Display

Zetong Zhang^a,#^, Yujie Chen^a,#^, Yuanyao Chen^a^, Leipeng Li^b^, Yonggang Wu^a^, Yanmin Yang^b^,*, and Hailei Zhang^a^,*

^a^College of Chemistry & Materials Science, Hebei University, 180 Wusi Road, 071002 Baoding, China;

^b^College of Physics Science and Technology, Hebei University, 180 Wusi Road, 071002 Baoding, China.

E-mails: mihuyym@163.com (Y. Y.); zhanghailei@hbu.edu.cn (H.Z.)

**Experimental**

**Materials**

Sm(NO_3_)_3_·6H_2_O, Lu(NO_3_)_3_·6H_2_O, Tb(NO_3_)_3_·6H_2_O, and 1,4-phenyldiboric acid were purchased from Shanghai Aladdin Biochemical Technology Co., Ltd. Oleic acid and citric acid were obtained from 3AMaterials®. NH_4_F was purchased from Shanghai Macklin Biochemical Co., Ltd. NaF was obtained from Tianjin Hengxing Chemical Reagent Co., Ltd. NaNO_3_ was purchased from Tianjin Kaitong Chemical Reagent Co., Ltd. Polyvinyl alcohol (PVA, *M*_n_ = 1750 ± 50 g/mol) was obtained from Sinopharm Chemical Reagent Co., Ltd. Distilled water was used throughout this study.

**Characterizations**

The X-ray-excited radioluminescence (RL) spectra were recorded using an Andor SR-500i spectrometer (Andor Technology Co. Belfast, UK) equipped with a Hamamatsu R928 photomultiplier.

X-ray photoelectron spectroscopy (XPS) was carried out on a Thermo Scientific ESCALab Xi^+^ (Thermo Fisher Scientific, US).

Powder X-ray diffraction (PXRD) patterns were recorded on a D8 ADVANCE X-ray powder diffractometer system (Bruker Corporation, German) using a tube voltage of 40 kV, a current of 40 mA, a range of 10° to 90° and a step size of 0.06°.

The morphological charateriations of the hydrogels were performed and recorded on a JEOL Ltd. JSM-7500F Cryo field emission scanning electron microscopy (SEM).

The dynamic light scattering (DLS) measurements were performed by using a commercialized spectrometer from Brookhaven BI-200SM Goniometer equipping a 17 mW He−Ne laser (633 nm). A Laplace inversion program was used to process the data to obtain the effective diameter and polydispersity index (PDI).

Stress-strain measurements were performed on a universal testing machine (YG-DB-50N, Dongguan Yuegao Electromechanical Equipment Technology Co., Ltd.) equipped with pneumatic fixtures. The tensile measurements were performed using a pulling speed of 10 mm min^-1^ until sample failure. The stress σ was recorded as a function of strain ε.

AR2000ex-type rotational rheometer (TA, US) was used to investigate the rheological properties.

Olympus DP 72 microscope camera was used to monitor the self-healing behavior.

**Synthesis and Preparation**

***α*-NaLuF₄:1%Sm³⁺**

NaNO₃ (0.145 g, 1.7 mmol), Lu(NO₃)₃·6H₂O (0.789 g, 1.683 mmol), and Sm(NO₃)₃·6H₂O (0.008 g, 0.017 mmol) were dissolved in 10 mL water and then added to 10 mL aqueous solution containing citric acid (0.038 g, 0.2 mmol). NaF (0.185 g, 4.4 mmol) was added into the system, and then the pH value was adjusted to 5.0 by the addition of nitric acid. The system was stirred at a low speed of 200 rpm for 30 min to achieve a homogeneous suspension. The agitation was continued for another 30 min. The obtained suspension was introduced into a hydrothermal reactor with a filling rate of 70% and then heated at 190 °C for 24 h. After cooling to room temperature, the solution was added to 200 mL absolute ethanol. The residue was collected by centrifugation and then washed with water and ethanol. After drying in a vacuum, the product *α*-NaLuF₄:1%Sm³⁺ was obtained as a white solid.

***α*-NaLuF₄:3%Sm³⁺**

NaNO₃ (0.145 g, 1.7 mmol), Lu(NO₃)₃·6H₂O (0.773 g, 1.649 mmol), and Sm(NO₃)₃·6H₂O (0.023 g, 0.051 mmol) were dissolved in 10 mL water and then added to 10 mL aqueous solution containing citric acid (0.038 g, 0.2 mmol). NaF (0.185 g, 4.4 mmol) was added into the system, and then the pH value was adjusted to 5.0 by the addition of nitric acid. The system was stirred at a low speed of 200 rpm for 30 min to achieve a homogeneous suspension. The agitation was continued for another 30 min. The obtained suspension was introduced into a hydrothermal reactor with a filling rate of 70% and then heated at 190 °C for 24 h. After cooling to room temperature, the solution was added to 200 mL absolute ethanol. The residue was collected by centrifugation and then washed with water and ethanol. After drying in a vacuum, the product *α*-NaLuF₄:3%Sm³⁺ was obtained as a white solid.

***α*-NaLuF₄:5%Sm³⁺**

NaNO₃ (0.145 g, 1.7 mmol), Lu(NO₃)₃·6H₂O (0.757 g, 1.615 mmol), and Sm(NO₃)₃·6H₂O (0.038 g, 0.085 mmol) were dissolved in 10 mL water and then added to 10 mL aqueous solution containing citric acid (0.038 g, 0.2 mmol). NaF (0.185 g, 4.4 mmol) was added into the system, and then the pH value was adjusted to 5.0 by the addition of nitric acid. The system was stirred at a low speed of 200 rpm for 30 min to achieve a homogeneous suspension. The agitation was continued for another 30 min. The obtained suspension was introduced into a hydrothermal reactor with a filling rate of 70% and then heated at 190 °C for 24 h. After cooling to room temperature, the solution was added to 200 mL absolute ethanol. The residue was collected by centrifugation and then washed with water and ethanol. After drying in a vacuum, the product *α*-NaLuF₄:5%Sm³⁺ was obtained as a white solid.

***α*-NaLuF₄:10%Sm³⁺**

NaNO₃ (0.145 g, 1.7 mmol), Lu(NO₃)₃·6H₂O (0.718 g, 1.530 mmol), and Sm(NO₃)₃·6H₂O (0.075 g, 0.170 mmol) were dissolved in 10 mL water and then added to 10 mL aqueous solution containing citric acid (0.038 g, 0.2 mmol). NaF (0.185 g, 4.4 mmol) was added into the system, and then the pH value was adjusted to 5.0 by the addition of nitric acid. The system was stirred at a low speed of 200 rpm for 30 min to achieve a homogeneous suspension. The agitation was continued for another 30 min. The obtained suspension was introduced into a hydrothermal reactor with a filling rate of 70% and then heated at 190 °C for 24 h. After cooling to room temperature, the solution was added to 200 mL absolute ethanol. The residue was collected by centrifugation and then washed with water and ethanol. After drying in a vacuum, the product *α*-NaLuF₄:10%Sm³⁺ was obtained as a white solid.

***α*-NaLuF₄:15%Sm³⁺**

NaNO₃ (0.145 g, 1.7 mmol), Lu(NO₃)₃·6H₂O (0.678 g, 1.445 mmol), and Sm(NO₃)₃·6H₂O (0.113 g, 0.255 mmol) were dissolved in 10 mL water and then added to 10 mL aqueous solution containing citric acid (0.038 g, 0.2 mmol). NaF (0.185 g, 4.4 mmol) was added into the system, and then the pH value was adjusted to 5.0 by the addition of nitric acid. The system was stirred at a low speed of 200 rpm for 30 min to achieve a homogeneous suspension. The agitation was continued for another 30 min. The obtained suspension was introduced into a hydrothermal reactor with a filling rate of 70% and then heated at 190 °C for 24 h. After cooling to room temperature, the solution was added to 200 mL absolute ethanol. The residue was collected by centrifugation and then washed with water and ethanol. After drying in a vacuum, the product *α*-NaLuF₄:15%Sm³⁺ was obtained as a white solid.

***α*‑NaLuF_4_**

NaNO_3_ (0.145 g, 1.7 mmol)，Lu(NO_3_)_3_·6H_2_O (0.797 g, 1.7 mmol) were dissolved in 10 mL water and then added to 10 mL aqueous solution containing citric acid (0.038 g, 0.2 mmol). NaF (0.185 g, 4.4 mmol) was added into the system, and then the pH value was adjusted to 5.0 by dropwise addition of nitric acid. The system was stirred at a low speed of 200 rpm for 30 min to form a homogeneous suspension, followed by continuous stirring for another 30 min. The obtained suspension was transferred into a hydrothermal reactor with a filling rate of 70% and then heated at 190 °C for 24 h. After cooling to room temperature, the resulting solution was poured into 200 mL absolute ethanol. The precipitate was collected by centrifugation and washed with water and ethanol. After drying in a vacuum, the product *α*‑NaLuF_4_ can be obtained as a white solid.

***β*-NaLuF₄:0.3%Sm³⁺**

NaOH (0.160 g, 4.0 mmol) was dissolved in 12 mL ethanol. Oleic acid (18 mL, 56.7 mmol) was added dropwise to the NaOH solution and then stirred for 0.5 h. NH₄F (0.37 g, 10 mmol), Sm(NO₃)₃·6H₂O (0.0013 g, 0.003 mmol), and Lu(NO₃)₃·6H₂O (0.4676 g, 0.997 mmol) were added to the mixture, and stirred for another 0.5 h, followed by transferring into a hydrothermal synthesis reactor equipped with a polytetrafluoroethylene liner. The solution was then heated at 190 °C for 24 h. The residue was collected by centrifugation and then washed with water, ethanol, and acetone. After drying in a vacuum, the product *β*-NaLuF₄:0.3%Sm³⁺ was obtained as a white solid.

***β*-NaLuF₄:0.5%Sm³⁺**

NaOH (0.160 g, 4.0 mmol) was dissolved in 12 mL ethanol. Oleic acid (18 mL, 56.7 mmol) was added dropwise to the NaOH solution and then stirred for 0.5 h. NH₄F (0.37 g, 10 mmol), Sm(NO₃)₃·6H₂O (0.0022 g, 0.005 mmol), and Lu(NO₃)₃·6H₂O (0.4667 g, 0.995 mmol) were added to the mixture, and stirred for another 0.5 h, followed by transferring into a hydrothermal synthesis reactor equipped with a polytetrafluoroethylene liner. The solution was then heated at 190 °C for 24 h. The residue was collected by centrifugation and then washed with water, ethanol, and acetone. After drying in a vacuum, the product *β*-NaLuF₄:0.5%Sm³⁺ was obtained as a white solid.​

***β*-NaLuF₄:1%Sm³⁺**

NaOH (0.160 g, 4.0 mmol) was dissolved in 12 mL ethanol. Oleic acid (18 mL, 56.7 mmol) was added dropwise to the NaOH solution and then stirred for 0.5 h. NH₄F (0.37 g, 10 mmol), Sm(NO₃)₃·6H₂O (0.0044 g, 0.010 mmol), and Lu(NO₃)₃·6H₂O (0.4643 g, 0.990 mmol) were added to the mixture, and stirred for another 0.5 h, followed by transferring into a hydrothermal synthesis reactor equipped with a polytetrafluoroethylene liner. The solution was then heated at 190°C for 24 h. The residue was collected by centrifugation and then washed with water, ethanol, and acetone. After drying in a vacuum, the product *β*-NaLuF₄:1%Sm³⁺ was obtained as a white solid.

***β*-NaLuF₄:3%Sm³⁺**

NaOH (0.160 g, 4.0 mmol) was dissolved in 12 mL ethanol. Oleic acid (18 mL, 56.7 mmol) was added dropwise to the NaOH solution and then stirred for 0.5 h. NH₄F (0.37 g, 10 mmol), Sm(NO₃)₃·6H₂O (0.0133 g, 0.030 mmol), and Lu(NO₃)₃·6H₂O (0.4549 g, 0.970 mmol) were added to the mixture, and stirred for another 0.5 h, followed by transferring into a hydrothermal synthesis reactor equipped with a polytetrafluoroethylene liner. The solution was then heated at 190 °C for 24 h. The residue was collected by centrifugation and then washed with water, ethanol, and acetone. After drying in a vacuum, the product *β*-NaLuF₄:3%Sm³⁺ was obtained as a white solid.

***β*-NaLuF₄:5%Sm³⁺**

NaOH (0.160 g, 4.0 mmol) was dissolved in 12 mL ethanol. Oleic acid (18 mL, 56.7 mmol) was added dropwise to the NaOH solution and then stirred for 0.5 h. NH₄F (0.37 g, 10 mmol), Sm(NO₃)₃·6H₂O (0.0222 g, 0.050 mmol), and Lu(NO₃)₃·6H₂O (0.4456 g, 0.950 mmol) were added to the mixture, and stirred for another 0.5 h, followed by transferring into a hydrothermal synthesis reactor equipped with a polytetrafluoroethylene liner. The solution was then heated at 190 °C for 24 h. The residue was collected by centrifugation and then washed with water, ethanol, and acetone. After drying in a vacuum, the product *β*-NaLuF₄:5%Sm³⁺ was obtained as a white solid.

***β*-NaLuF₄:10%Sm³⁺**

NaOH (0.160 g, 4.0 mmol) was dissolved in 12 mL ethanol. Oleic acid (18 mL, 56.7 mmol) was added dropwise to the NaOH solution and then stirred for 0.5 h. NH₄F (0.37 g, 10 mmol), Sm(NO₃)₃·6H₂O (0.0444 g, 0.100 mmol), and Lu(NO₃)₃·6H₂O (0.4221 g, 0.900 mmol) were added to the mixture, and stirred for another 0.5 h, followed by transferring into a hydrothermal synthesis reactor equipped with a polytetrafluoroethylene liner. The solution was then heated at 190 °C for 24 h. The residue was collected by centrifugation and then washed with water, ethanol, and acetone. After drying in a vacuum, the product *β*-NaLuF₄:10%Sm³⁺ was obtained as a white solid.

***β*-NaLuF₄:15%Sm³⁺**

NaOH (0.160 g, 4.0 mmol) was dissolved in 12 mL ethanol. Oleic acid (18 mL, 56.7 mmol) was added dropwise to the NaOH solution and then stirred for 0.5 h. NH₄F (0.37 g, 10 mmol), Sm(NO₃)₃·6H₂O (0.0666 g, 0.150 mmol), and Lu(NO₃)₃·6H₂O (0.3987 g, 0.850 mmol) were added to the mixture, and stirred for another 0.5 h, followed by transferring into a hydrothermal synthesis reactor equipped with a polytetrafluoroethylene liner. The solution was then heated at 190 °C for 24 h. The residue was collected by centrifugation and then washed with water, ethanol, and acetone. After drying in a vacuum, the product *β*-NaLuF₄:15%Sm³⁺ was obtained as a white solid.

***β*-NaLuF_4_**

NaOH (0.160 g, 4.0 mmol) was dissolved in 12 mL ethanol. Oleic acid (18 mL, 56.7 mmol) was added dropwise to the NaOH solution and then stirred for 0.5 h. NH_4_F (0.37 g, 10 mmol), Lu(NO_3_)_3_·6H_2_O (0.469 g, 1.00 mmol) were added to the mixture, and stirred for another 0.5 h, followed by transferring into to hydrothermal synthesis reactor equipped with polytetrafluoroethylene liner. The solution was then heated at 190 °C for 24 h. The residue was collected by certification and then washed with water, ethanol, and acetone. After drying in a vacuum, the product *β*-NaLuF_4_ can be obtained as a white solid.

**Surface modification**

Take oleic acid (OA)-modified *β*-NaLuF_4_:3%Sm^3+^ as an example. 1.0 g *β*-NaLuF_4_:3%Sm^3+^ was added into 7.5 mL OA and heated at 80 °C for 1 h. After cooling to room temperature, the residue was collected by centrifugation and then washed with ethanol and acetone. The obtained solid was dried in the vacuum to afford OA@*β*-NaLuF_4_:3%Sm^3+^ as a gray solid. Other OA-modified nanoparticles can also be prepared according to the above-mentioned procedure.

**Hydrogels**

**Poly(vinylalcohol-*co*-1,4-phenyldiboric acid) hydrogel (Gel-0)**

PVA (1.35 g) was dissolved in 12.0 mL distilled water at 80 °C. Then, the 1,4-phenyldiboric acid-containing NaOH solution (pH = 10.5) was added to the PVA solution. The system was stirred for about 5 min, then transferred to the mold. The hydrogel was stored at -20 °C for 2 h and thawed at room temperature to afford H1.

**Phosphor-incorporated hydrogels (Gel-1, Gel-2, Gel-3 and Gel-4)**

Take OA@*β*-NaLuF_4_:3%Sm^3+^-incorporated poly(vinylalcohol-*co*-1,4-phenyldiboric acid) hydrogel (H2) as an example. OA@*β*-NaLuF_4_:3%Sm^3+^ (200 mg) and PVA (1.35 g) were dissolved in 12.0 mL distilled water at 80 °C. Then the 1,4-phenyldiboric acid -containing NaOH solution (pH = 10.5) was added into the PVA solution. The system was stirred for about 5 min then transferred to the mold. The hydrogel was stored at -20 °C for 2 h and thaw at room temperature to afford Gel-1. HNTs@YF_3_:Tb^3+^-incorporated hydrogel-incorporated poly(vinylalcohol-*co*-1,4-phenyldiboric acid) hydrogel (Gel-2) was prepared according to the above-mentioned procedure. Gel-3 is a hybrid hydrogel doped with different feed ratios between HNTs@*α*-NaLuF_4_:Tb^3+^ and OA@*β*-NaLuF_4_:3%Sm^3+^. Gel-4 was prepared by varying the feed ratio between HNTs@*α*-NaLuF_4_:Tb^3+^ and OA@*α*-NaLuF_4_:3%Sm^3+^.

**Additional data**


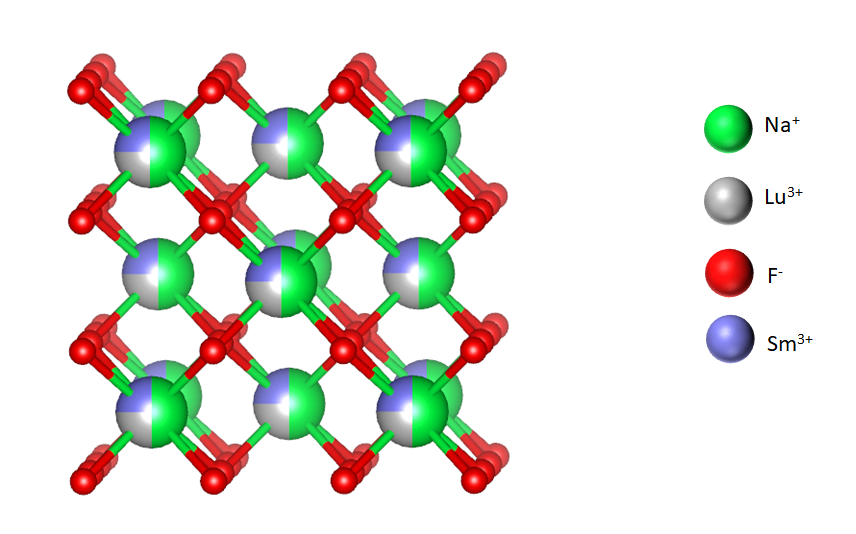


**Figure S1.** Unit cell diagram of cubic *α*-NaLuF_4_:*x*Sm^3+^


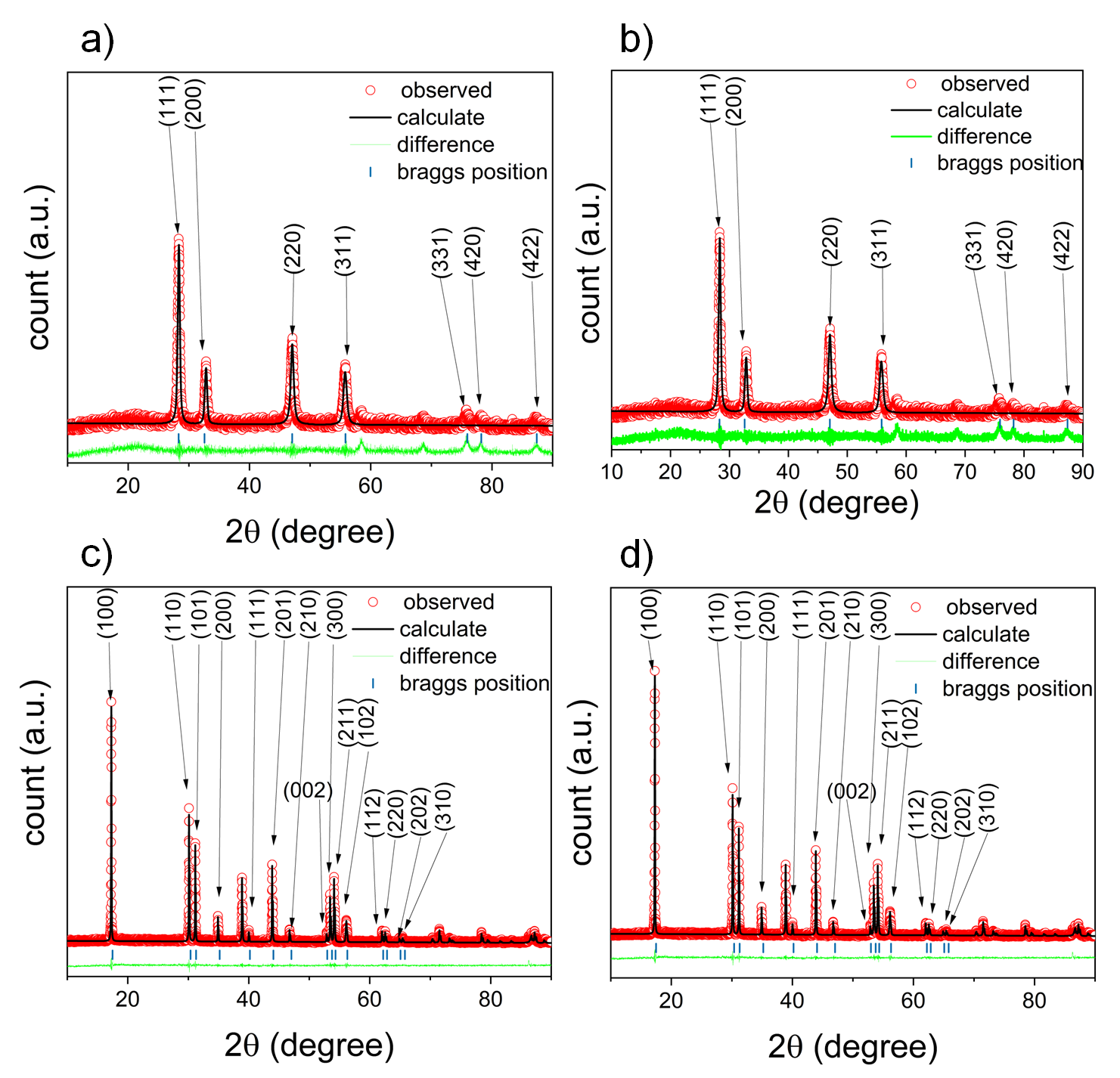


**Figure S2.** Rietveld refinement of both pure and Sm³⁺-doped NaLuF₄ samples. a) Pure *α*‑NaLuF_4_; b) *α*-NaLuF₄:3%Sm³⁺; c) Pure *β*‑NaLuF_4_; b) *β*-NaLuF₄:3%Sm³⁺


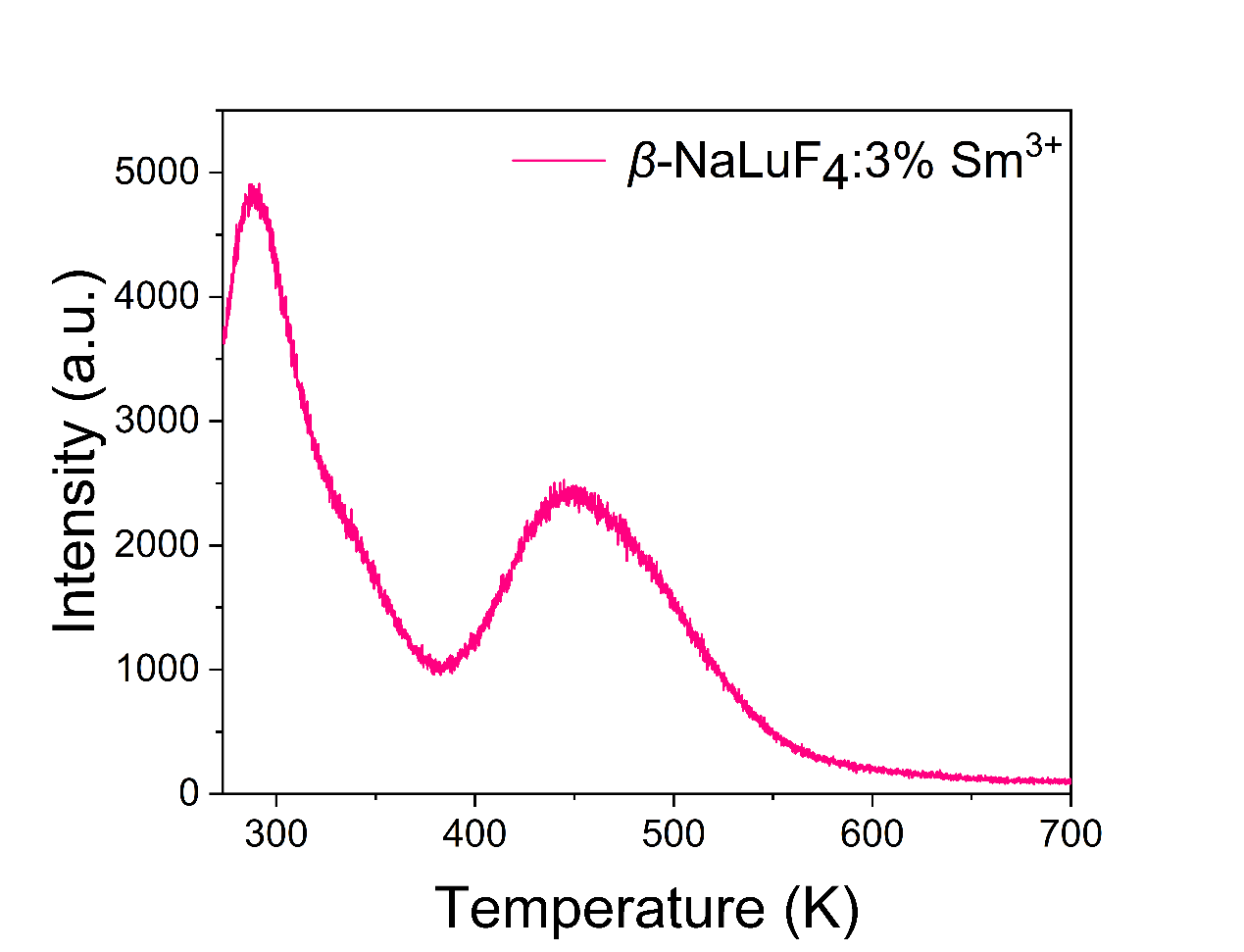


**Figure S3.** Thermoluminescence (TL) spectrum of *β*-NaLuF₄:3%Sm³⁺ recorded at 598 nm at a heating rate of 5 °C/min


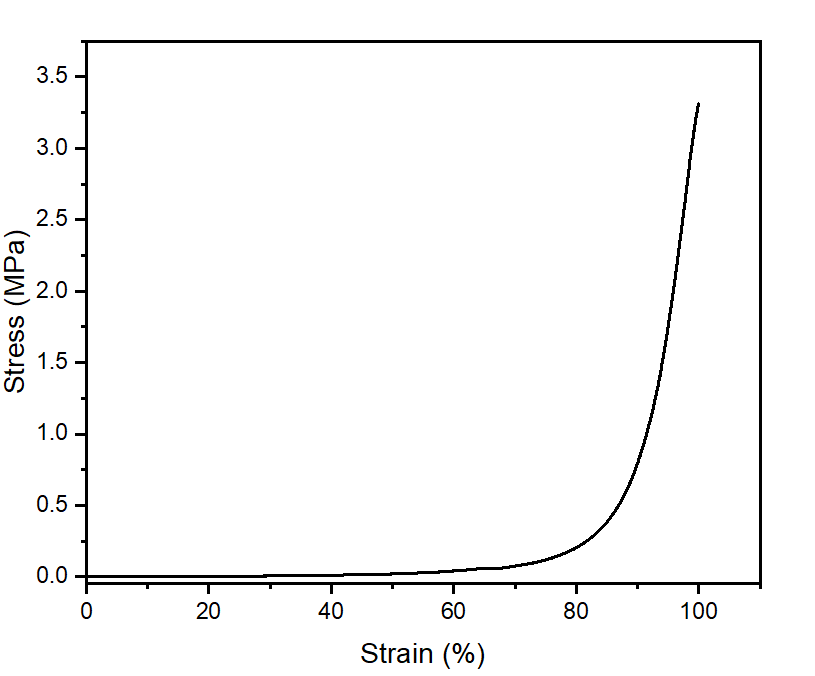


**Figure S4.** Stress-strain curve of Gel-1 during compressive process


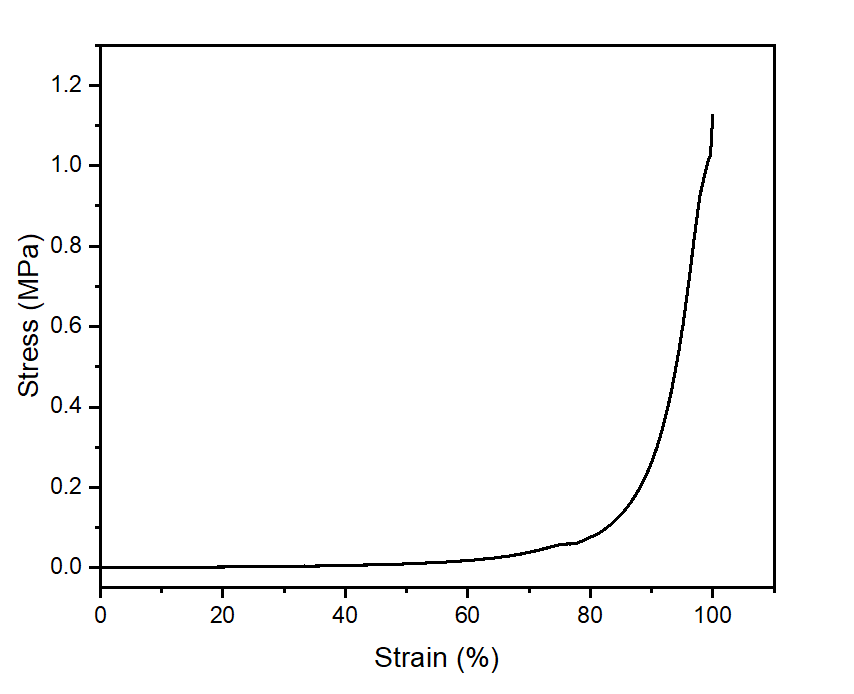


**Figure S5.** Stress-strain curve of Gel-0 during compressive process


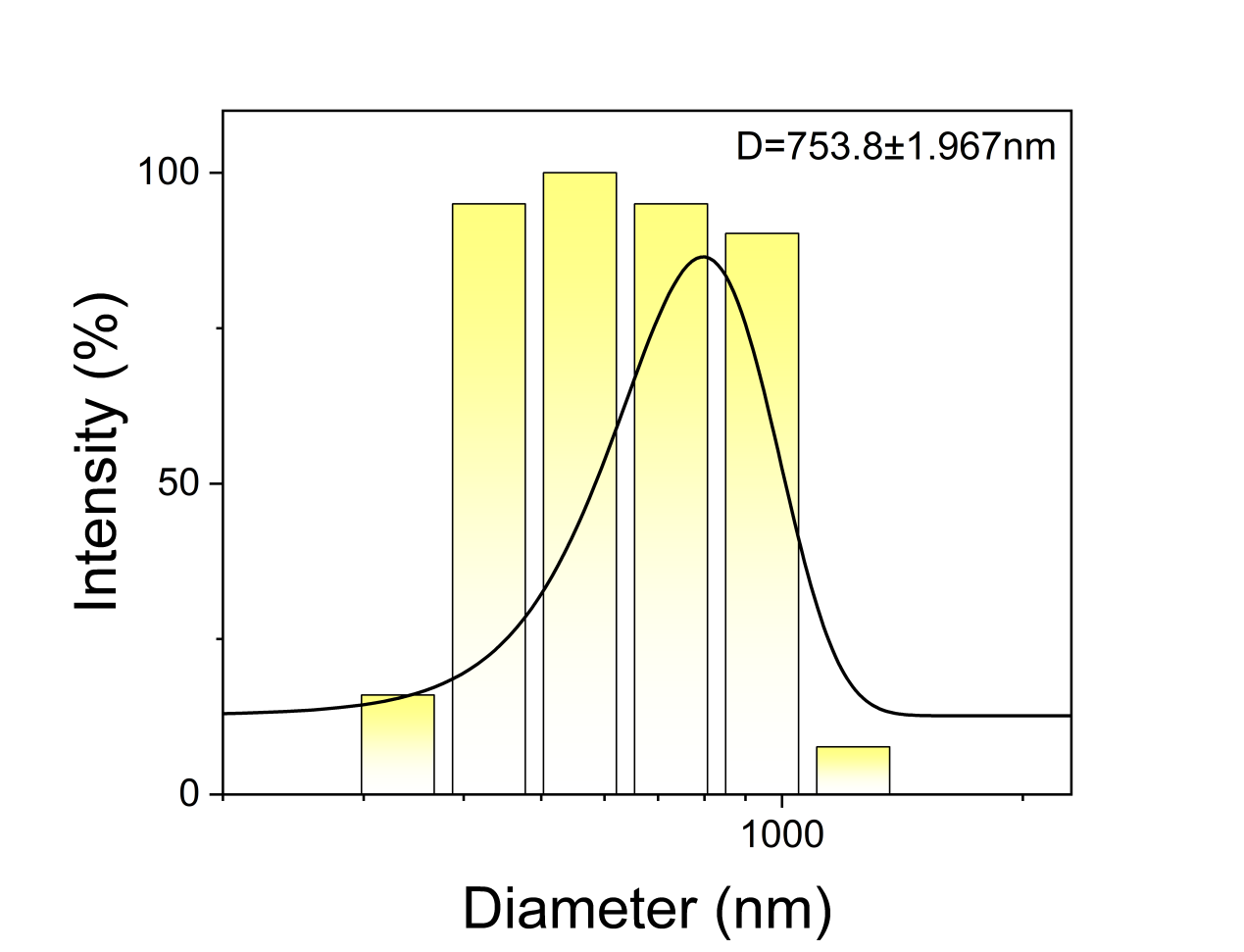


**Figure S6.** Size distribution of OA@*α*-NaLuF_4_:3%Sm^3+^ in aqueous phase.
